# Granulomatous Lung Disease Requiring Mechanical Ventilation Induced by a Single Application of Oxaliplatin-Based Chemotherapy for Colorectal Cancer: A Case Report

**DOI:** 10.1155/2013/683948

**Published:** 2013-04-08

**Authors:** Dane Wildner, Frank Boxberger, Axel Wein, Kerstin Wolff, Heinz Albrecht, Gudrun Männlein, Rolf Janka, Kerstin Amann, Jürgen Siebler, Werner Hohenberger, Markus F. Neurath, Richard Strauß

**Affiliations:** ^1^Department of Medicine 1, University Hospital Erlangen, Ulmenweg 18, 91054 Erlangen, Germany; ^2^Radiological Institute, University Hospital Erlangen, Maximiliansplatz 1, 91054 Erlangen, Germany; ^3^Institute of Pathology, University Hospital Erlangen, Krankenhausstraße 12, 91054 Erlangen, Germany; ^4^Department of Surgery, University Hospital Erlangen, Krankenhausstraße 12, 91054 Erlangen, Germany

## Abstract

Combined chemotherapeutic regimens in conjunction with oxaliplatin are considered safe and effective treatment options in the clinical management of metastatic colorectal cancer. A 62-year-old male patient with a metastatic rectal carcinoma developed a pulmonary reaction after the first application of the combined standard chemotherapy regimen (5-fluorouracil and sodium folinic acid as a 24 h infusion and oxaliplatin). Following the first dose of chemotherapy, the patient developed acute dyspnoea and fever. A computerised scan of the chest revealed bilateral pulmonary patchy consolidation. Despite high-dose empiric antibiotic and antimycotic treatment, no clinical improvement was seen. The patient's condition deteriorated, and he required invasive mechanical ventilation. Diagnostic thoracoscopic wedge resections were performed for further diagnosis. The histological workup revealed distinct granulomatous inflammation, but no microbial pathogens were to be found. Thereupon, a drug-induced reaction to chemotherapy was suspected and high-dose steroid treatment initiated. Subsequently, the patient's respiratory condition improved and he was extubated. The present case exemplifies the rare course of a bilateral pneumonia-like, drug-induced granulomatous reaction following a single application of oxaliplatin. In addition to the known side effects of oxaliplatin-containing combination chemotherapy, unexpected serious adverse events in the form of pulmonary toxicities should also be taken into account.

## 1. Introduction 

The standard palliative treatment of patients with metastatic colorectal cancer involving the application of 5-fluorouracil-(5-FU-) based chemotherapy combined with irinotecan or oxaliplatin in first- and second-line treatment extended patient survival by up to 22 months [1–3]. In addition, innovative molecular approaches such as antiangiogenesis or inhibition of signal epidermal growth factor receptor transmission have become more important [4, 5]. Currently, the interdisciplinary management of colon cancer patients who initially do not respond to curative therapy is at the focus of efforts to prolong survival and maintain quality of life. In some cases, a secondary metastatic resection of primarily unresectable metastases after downstaging offers a curative option. A necessary proviso, however, is close interdisciplinary cooperation on the part of all those involved [6, 7].

The occurrence of adverse events during chemotherapy makes it necessary to modify or, in rare cases, even to discontinue treatment. Depending on the treatment regimen applied, the most frequent serious side effects that may occur following the use of oxaliplatin (in combination with) 5-FU and folinic acid are haematological (13%–52%), gastrointestinal (10%–33%), and neurological (0%–8%) toxicities. Although most of these reactions can be readily controlled, in rare cases they may necessitate discontinuation of treatment. In comparison with the high level of oxaliplatin usage, pulmonary toxicity is very rare [8–10].

## 2. Case Presentation

A 62-year-old male patient attended the outpatient department for endoscopic clarification of lower gastrointestinal bleeding haemorrhage. Since the patient had atrial fibrillation, he was on phenprocoumon. Colonoscopy revealed a circular exulcerating carcinoma located in the upper third of the rectum. Histological workup revealed an adenocarcinoma. Ultrasonography of the abdomen showed a number of virtually hypoechoic lesions compatible with liver metastases. Consequently, deep anterior rectal resection plus peritonectomy in the region of the posterior wall of the bladder combined with a descendorectostomy were performed to resolve a high-grade stenosis caused by the rectal carcinoma. Intraoperatively, peritoneal carcinomatosis was also found. The findings were categorized pathohistologically as pT4b, pN2 (5/28), L0, V1, pM1 (PER, HEP), and G2 (UICC Stage IV). The postoperative course was uneventful. Diffuse, nonresectable liver metastases and peritoneal carcinomatosis were diagnosed, and palliative treatment with combination chemotherapy comprising high-dose 5-FU and sodium folinic acid in the form of a 24 h infusion plus oxaliplatin every second week was scheduled [7].

**Figure 1:**
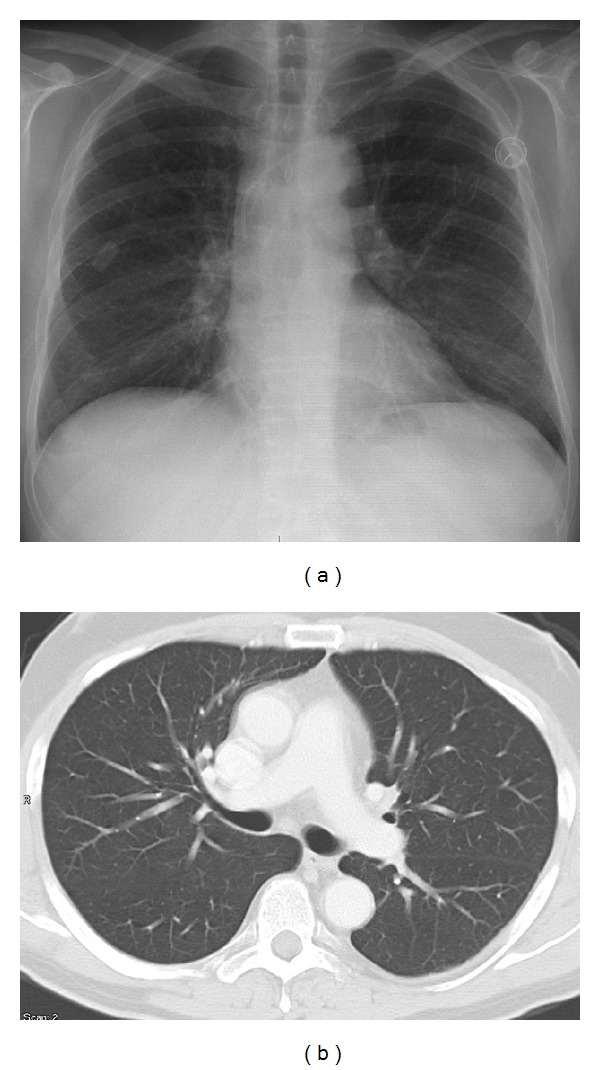
X-ray (one day after inpatient admittance) and CT scan of the chest (one month prior to first chemotherapy administration). Normal finding in both lungs.

**Figure 2:**
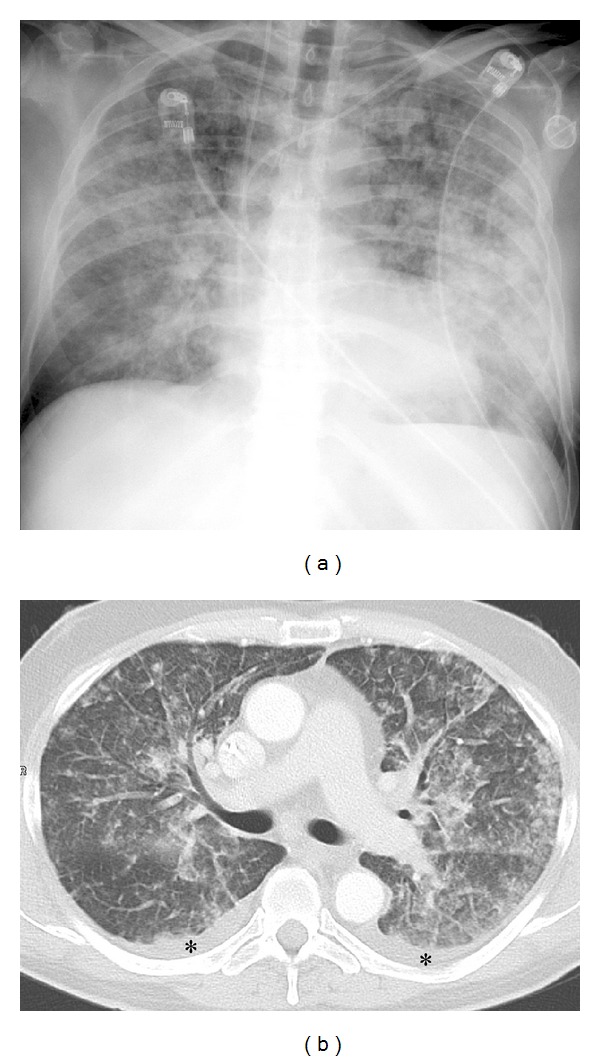
X-ray (seven days after inpatient admittance) and CT scan of the chest (five days after initiation of chemotherapeutic treatment). Patchy airspace consolidation with peribronchial and peripheral distribution. Small pleural effusion bilaterally (black asterisk).

**Figure 3:**
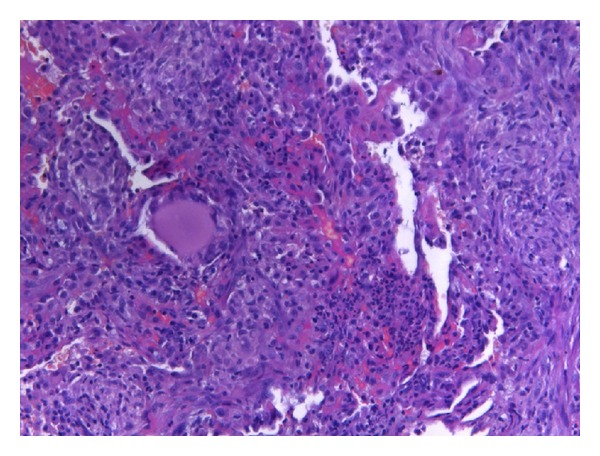
Lung biopsy specimen obtained by video-assisted thoracoscopic wedge resection (HE ×10). Extensive granulomatous inflammation. No evidence of fibrosis or malignancy.

One day after receiving the first dose (5-FU 2.000 mg/m^2^, sodium folinic acid 500 mg/m^2^ combined with a previous application of oxaliplatin 85 mg/m^2^) the patient presented at our emergency unit with progressive dyspnoea and a subfebrile temperature (38.4°C). Laboratory markers of inflammation were elevated (leucocytes 12.400/*μ*L (normal range: 4.000–10.000/*μ*L), CRP 41 mg/L (normal range < 5 mg/L)). Empirical antibiotic treatment with ampicillin/sulbactam was initiated. At that time the conventional chest X-ray and the previous computerised tomography of the chest (obtained one month prior to the initiation of chemotherapy) were inconspicuous (Figure 1). Nevertheless, the patient's condition continued to deteriorate. On the patient's third day in hospital, his antibiotic therapy was adjusted to piperacillin/sulbactam and ciprofloxacin. From the fifth day onwards, fluconazole was added. During the course of treatment, both the conventional X-ray and the CT scan of the chest revealed bilateral patchy consolidations of both lungs and low-grade pleural effusions (Figure 2). After seven days, the patient was transferred to the intensive care unit with respiratory failure (O_2_-saturation 70%). Antibiotic treatment was continued, and noninvasive mechanical ventilation initiated. On the following day, however, the patient had to be intubated because of progressive respiratory distress (arterial blood gases: FiO_2_ 0.55, paO_2_ 36.2 mmHg, pCO_2_ 46.7 mmHg, Horowitz-Index 65.8). Bronchoalveolar lavage was performed. Neither microbiological nor virological examinations revealed the presence of pathogens. Ten days after intubation, the patient's gas exchange parameters showed no signs of clinical improvement (arterial blood gases: FiO_2_ 1.0, paO_2_ 99 mmHg, pCO_2_ 43.0 mmHg, Horowitz-Index 99). The laboratory markers of inflammation continued to increase (maximum values: leucocytes 15.500/*μ*L (normal range: 4.000–10.000/*μ*L), CRP 174 mg/L (normal range < 5 mg/L)). Antibiotic medication was temporarily discontinued, and thoracoscopic wedge resections were performed in all three right pulmonary lobes. Prior to the availability of the microbiological and histological results, antibiotic treatment with ceftazidime and fosfomycin was applied. Neither the Grocott nor the Ziehl-Neelsen or Auramine staining revealed evidence of bacterial or mycotic pathology. Both the mycobacterial PCR and the bacteriological cultures were negative. Repeated microbiological and viral examinations (bronchoalveolar lavage, bloodcultures, galactomannan assay, central venous catheter tip, urine, and legionella-antigens in the urine) all remained negative. No signs of systemic autoimmune disease were to be found. The histological findings revealed extensive granulomatous inflammation with no evidence of malignancy (Figure 3). No eosinophilic infiltration, fibroinflammatory buds, or collagen bundles as evidence of a hypersensitivity reaction or pulmonary fibrosis were seen. Since a drug-induced lung reaction to chemotherapy was suspected, i.v. high-dose steroid treatment (initial application: 1 g prednisolone per day) was started. Within 36 hours, the gas exchange parameters improved, and the patient was extubated after 4 days on steroid treatment and 16 day**s **on invasive mechanical ventilation. During the following days, intermittent noninvasive mechanical ventilation was gradually reduced and finally discontinued on the third postextubation day. The daily prednisolone dose was reduced to 70 mg after eight days, to 50 mg after 12 days, and finally tapered off over 3 weeks.

**Figure 4:**
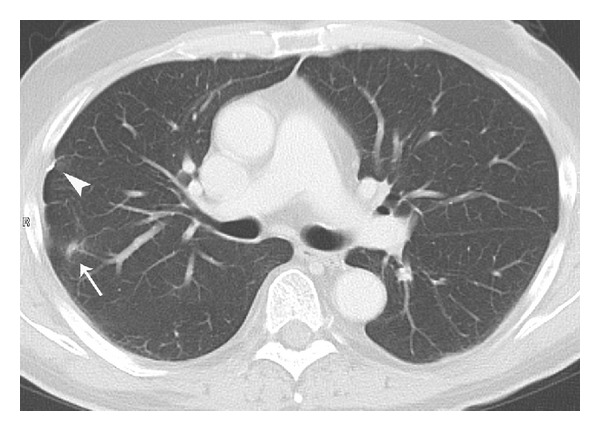
CT scan of the chest five months after the first application of chemotherapy. Small scar in the right upper lobe (white arrow) and small pleural calcification (white arrowhead) as residual lesions of the inflammatory process.

On discharging the patient from the inpatient department, we reevaluated his clinical condition and radiological findings and discussed his treatment options with him in detail. His general state of health (ECOG 1) and alimentary status were both good. A dihydropyrimidine-dehydrogenase deficiency, which is sometimes associated with high-grade 5-FU toxicity, was excluded. A CT scan of the chest 5 months after respiratory failure (Figure 4) showed virtually complete resolution with only slight residual subpleural scarring and pleural calcifications.

Finally, with the informed consent of the patient, we decided to implement palliative second-line treatment (5-FU and sodium folinic acid in the form of a 24 h infusion plus weekly irinotecan) [11]. All in all, the patient received three cycles of palliative second-line treatment, one cycle of palliative third-line treatment (5-FU and sodium folinic acid in the form of a 24 h infusion combined with weekly irinotecan and cetuximab), one cycle of palliative fourth-line treatment (5-FU and sodium folinic acid in the form of a 24 h infusion combined with irinotecan, and bevacizumab every two weeks), and finally additional internal radiotherapy (SIRT). The additional palliative treatment regimens were well tolerated, and no more serious toxic side effects occurred. Pulmonary symptoms did not reoccur. From initiation of palliative first-line treatment, the patient survived 21 months. He died of tumour-associated cardiocirculatory failure caused by progression of the liver metastases.

## 3. Discussion

Acute lung injury after combination chemotherapy including oxaliplatin does occasionally occur. In view of the frequency of such treatment, however, reports of acute respiratory deterioration (sometimes taking a fatal course) associated with the use of oxaliplatin are rare, and only a few publications describe pulmonary fibrosis, hypersensitivity reactions, or organizing pneumonia [12–25]. Drug-induced pneumonitis is usually a diagnosis of exclusion. Symptoms are nonspecific and may include dyspnoea, fever, or respiratory failure. Infections, alveolar haemorrhage, lymphangiosis, and heart failure are the main differential diagnoses in cancer patients on chemotherapy who develop respiratory distress. In our patient, extensive diagnostic workup, which included blood, bronchoalveolar lavage, urine, and histological examinations, failed to produce a specific diagnosis. Retrospectively, no pathogens were ever detected, despite extensive diagnostic measures during the period of hospitalisation, and the response to empirical broad-spectrum antibiotic treatment failed to bring about any consistent clinical improvement. A lung biopsy viathoracoscopic wedge resection in all three right pulmonary lobes was deemed necessary. The histological workup of all three lobes showed the same pattern of a granulomatous infiltration with no signs of pulmonary fibrosis or malignancy. This patient report is the first to describe acute lung injury with a distinct granulomatous reaction associated with this combination of drugs. No other causes of granulomatous lung disease (e.g. sarcoidosis, Wegener disease, or mycobacterial infection) were to be found. The rapid and sustained improvement achieved with high-dose steroid therapy in the absence of any signs of autoimmune disease permits the conclusion to be drawn that the inflammatory changes had been caused by a drug-induced pulmonary reaction.

Since the concomitant medication was unlikely to be responsible for the initial findings, the pathological course must have been caused by the oxaliplatin. After having survived the acute reaction, the patient underwent repeated treatment with palliative combination therapy with 5-FU and sodium folinic acid in addition to other agents in the second-, third-, and fourth line treatments. 

Reexposure to 5-FU and sodium folinic acid did not provoke any signs of pulmonary toxicity. We therefore conclude that the observed lung disease was triggered by the oxaliplatin alone or—less likely—in combination with 5-FU and sodium folinic acid.

Various agents have previously been reported to cause such pulmonary side effects as fibrosing alveolitis (e.g. bleomycin, busulfan, or trastuzumab) or granulomatous lung disease (e.g. methotrexate, BCG, TNF-*α* blocking agents, gefitinib, or everolimus) [26–32]. Usually the disorders develop slowly, and a continued worsening of unspecific symptoms (e.g. cough, dyspnea, or haemoptysis) can be found. These are often reversible after discontinuation of the suspected medication. In some cases, further diagnostic workup and steroid treatment has been necessary. Apart from the presumed involvement of immunological factors in the development of drug-induced pulmonary pathologies, a histological examination of the lung for possible infectious conditions (e.g. caseating granulomas in mycobacterial infections) may help to establish the differential diagnosis of suspected drug-induced lung disease. A drug-induced granulomatous lung reaction after a single administration of oxaliplatin has not so far been reported.

## 4. Conclusions

Clinical application of combined chemotherapy with oxaliplatin is considered a safe and efficient method of treating advanced colorectal cancer. Apart from the consideration of haematological, gastrointestinal, and neurological side effects, possible pulmonary toxicities should also be taken into account. Lung biopsy should be considered in otherwise unexplained pulmonary disease in patients with chemotherapy.

## Abbreviations

5-FU: 5-Fluorouracil
UICC: Union Internationale Contre le Cancer
CRP: C-reactive protein
PCR: Polymerase chain reaction
ECOG: Eastern cooperative oncology group
CT: Computed tomography
SIRT: Selective internal radiotherapy
BCG: Bacillus Calmette-Guérin
TNF-*α*: Tumour necrosis factor-alpha.
